# Modelling the Predictors of Mobile Health (mHealth) Adoption among Healthcare Professionals in Low-Resource Environments

**DOI:** 10.3390/ijerph20237112

**Published:** 2023-11-26

**Authors:** Mehreen Azam, Salman Bin Naeem, Maged N. Kamel Boulos, Anthony Faiola

**Affiliations:** 1 Department of Information Management, The Islamia University of Bahawalpur, Bahawalpur 63100, Pakistan; 2 School of Medicine, University of Lisbon, 1649-028 Lisbon, Portugal; 3 Department of Health and Clinical Sciences, College of Health Sciences, University of Kentucky, Lexington, KY 40506, USA

**Keywords:** mobile health, adoption, healthcare professionals, UTAUT, low-resource settings, electronic health

## Abstract

This study was conducted with objectives to measure and validate the unified theory of the acceptance and use of technology (UTAUT) model as well as to identify the predictors of mobile health (mHealth) technology adoption among healthcare professionals in limited-resource settings. A cross-sectional survey was conducted at the six public and private hospitals in the two districts (Lodhran and Multan) of Punjab, Pakistan. The participants of the study comprised healthcare professionals (registered doctors and nurses) working in the participating hospitals. The findings of the seven-factor measurement model showed that behavioral intention (BI) to mHealth adoption is significantly influenced by performance expectancy (β = 0.504, CR = 5.064, *p* < 0.05) and self-concept (β = 0.860, CR = 5.968, *p* < 0.05) about mHealth technologies. The findings of the structural equation model (SEM) showed that the model is acceptable (χ^2^ (df = 259) = 3.207; *p* = 0.000; CFI = 0.891, IFI = 0.892, TLI = 0.874, RMSEA = 0.084). This study suggests that the adoption of mHealth can significantly help in improving people’s access to quality healthcare resources and services as well as help in reducing costs and improving healthcare services. This study is significant in terms of identifying the predictors that play a determining role in the adoption of mHealth among healthcare professionals. This study presents an evidence-based model that provides an insight to policymakers, health organizations, governments, and political leaders in terms of facilitating, promoting, and implementing mHealth adoption plans in low-resource settings, which can significantly reduce health disparities and have a direct impact on health promotion.

## 1. Introduction

Mobile health (mHealth), a term coined by Robert Istepanian, refers to the use of mobile technologies and communication networks for the delivery of healthcare [1]. The World Health Organization defines mHealth as “the use of mobile devices, such as mobile phones, patient monitoring devices, personal digital assistants (PDAs), and other wireless devices, to support the practice of medicine and public health” [2]. The application of information and communication technology (ICT), particularly mobile technology, has altered healthcare service delivery and made it more widely accessible and less expensive. Patients can use mobile devices to access their medical records, lab results, medical imaging, and prescriptions in order to be informed of their diagnosis, disease control, and monitoring as well as to make an appointment with a doctor [3].

mHealth solutions address healthcare challenges such as the increase in communicable or non-communicable diseases and the burden of healthcare cost, and it can also empower patients and families to take preventive measures against diseases and to self-manage their health by providing access to healthcare information and services wherever they are needed [4]. As part of the e-health initiative, telemedicine services are accessible to the general public around the world. Some examples include Healthline in Bangladesh, HMRI in India, Teledoctor in Pakistan, Medical Home in Mexico, Fonemed in the United States, NHS Direct in the United Kingdom, Project REMOTE in Europe, and Project Masiluleke in South Africa [5]. mHealth solutions have the potential to prevent deaths from diseases that can be cured or controlled, such as malaria, asthma, and diarrhea, which are responsible for the death of large numbers of children every year. Major causes of these largely preventable deaths include a lack of knowledge and understanding of the healthcare system, geographical distance from healthcare facilities, limited access to healthcare services, and poverty [6,7].

mHealth is a low-cost and efficient technology for sharing knowledge on disease prevention, self-management, and diagnostics that aids in educating people in underdeveloped countries. It provides a practical and convenient way to spread preventive and awareness health messages [8]. Several studies have reported that the accessibility of mobile-based health information resources improve individuals’ knowledge of health in terms of different diseases, such as AIDS and HIV systems [9,10,11], oral contraceptives [12] quitting smoking [13], and pregnancy in women [14]. Additionally, the emergence of clinical decision support systems in underdeveloped countries is encouraging since they can help in diminishing the knowledge gap between patients and healthcare professionals. In Kenya, a malaria treatment adherence management trial system was implemented in 107 rural healthcare facilities. The system’s findings revealed that treatment adherence improved by 31.7% in the short term and 28.6% in the long term [15]. Clinical support systems through mHealth have been successfully implemented in several developing countries to monitor ECG [16] and child mortality [17]. mHealth also supports remote monitoring, provides a new pathway to treating patients, and improves survival rates in low-resource settings, where access to hospitals is limited and healthcare facilities are inadequate [18].

The present study is significant in terms of identifying the predictors that play a determining role in the adoption of mHealth among healthcare professionals. This study plays a significant role in presenting an evidence-based model that can suggest mHealth adoption predictors to policy makers, health organizations, government organizations, and political leaders in terms of facilitating, promoting, and implementing mHealth adoption plans in low-resource settings, which can significantly help to reduce health disparities and will have a direct impact on health promotion. Moreover, this study will bridge the gap in the inadequate literature on the predictors of mHealth adoption among healthcare professionals in rural areas and in areas where there is inadequate health infrastructure and resources.

### 1.1. Mobile Health Applications

mHealth applications are valuable due to the pre-existing infrastructure for mobile phones and a user community that is already familiar with the technology. They can provide a means of communication with generation Z, who have embraced mobile technology far more quickly than any other generation group. mHealth apps can help with healthcare delivery in a number of different ways. However, some programs simply advocate for digital health [19]. Others provide a range of healthcare services, including information, reminders for treatments, and data collection [20]. The types of services that are included in different categories are shown in Table 1 along with how the categories overlap [9,19,21,22,23,24,25].

In developing countries like Pakistan, mHealth has revolutionized healthcare delivery. mHealth includes applications designed for users of all ages and with a wide range of demands (as shown in Table 2).

### 1.2. Theoretical Framework and the Process of Hypotheses Development

Presently, in the scientific community, there is noteworthy academic and professional concern with the process of the implementation and utilization of information technology (IT) and information systems (IS). Researchers have established various theoretical frameworks and models from this particular standpoint during the last few decades, such as the theory of planned behavior [26], the diffusion of innovation theory [27], social cognitive theory [28], the motivational model [29], the model of personal computer utilization [30], innovation diffusion theory [31], the theory of reasoned action (TRA) [32], and the technology acceptance model (TAM). In 2003, Venkatesh et al. proposed the unified theory of acceptance and use of technology (UTAUT), which attempts to explain the process by which individuals adopt a particular technology [33]. The UTAUT model is a highly prevalent theoretical framework utilized in the field of technology adoption research and encompasses all relevant variables, taking into consideration the influence of eight prominent theories on the behavioral intention (BI) to adopt a technology, including TAM. The UTAUT model is widely recognized as an efficient framework for understanding the adoption of technology in various fields [34]. Furthermore, in contrast to the eight models upon which it is established, researchers have demonstrated that the UTAUT model has a significantly higher level of efficacy, up to 70%, in terms of describing users’ intentions [35,36].

**Figure 1:**
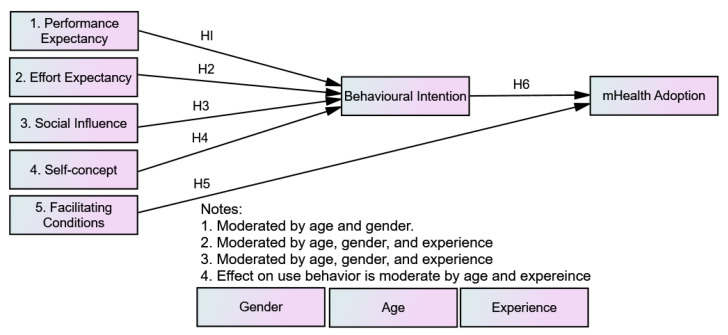
Conceptualization of hypotheses (H1—H6) development. H = Hypothesis.

Venkatesh et al. [33] analyzed the previously reported models on technology acceptance and identified the constructs that were significantly useful and/or integrated in the models or their extensions. The researchers eliminated overlapping concepts and finally developed a unified model with an overall inclusive explanatory power to conceptualize and predict individuals’ attitudes toward technology. Chib et al. [37] reviewed mHealth adoption studies in low-resource settings. Their analysis found a scarcity of research that provides theoretical insight into mHealth adoption. Thereby, the theoretical foundation of the present study is based on the unified theory of acceptance and use of technology (UTAUT) model [33]. Several researchers have used the integrated conceptual framework to theorize the adoption behavior of technology [33,38,39,40]. Our study’s conceptualization (as shown in Figure 1) is based on the UTAUT framework, suggesting that the adoption of mHealth is influenced by the determinants of UTAUT, i.e., performance expectancy (PE), effort expectancy (EE), facilitating conditions (FCs), social influence (SI), and behavioral intention (BI). However, we added an extra construct, self-concept (SC), to the determinants of the UTAUT model.

#### 1.2.1. Performance Expectancy (PE)

Venkatesh et al. (2003) [33] reported that performance expectancy (PE) is conceptualized as the extent to which a person considers that adopting a technology will enable him or her to increase their productivity at work. PE contains the characteristics of five theories, i.e., extrinsic motivation from the motivational model [29] perceived usefulness from TAM, task fit from the personal computer utilization theory, and relative advantage from social cognitive theory. In the context of mHealth, PE is conceptualized as the degree to which a healthcare professional believes that using mobile devices for healthcare services would be beneficial. Numerous research studies have predicted PE as a significant predictor of healthcare technology adoption [33,39,40,41,42,43].

**H1:**  *Performance expectancy (PE) has a statistically significant influence on the behavioral intention to adopt mHealth.*

#### 1.2.2. Effort Expectancy (EE)

Effort expectancy (EE) is described as the level of ease a healthcare professional feels while using mHealth. Three constructs of other theories represent EE, which are perceived ease of use (TAM, TAM2), complexity (personal computer utilization theory), and ease of use (innovation diffusion theory). Numerous empirical studies have demonstrated that EE directly influence users’ intentions to adopt new technologies [35,44]. EE was found as a predictor of users’ intent to use e-Health services, clinical decision support systems, and mHealth adoption [44,45,46] Therefore, it is hypothesized that:

**H2:**  *Effort expectancy (EE) has a statistically significant influence on the behavioral intention to adopt mHealth.*

#### 1.2.3. Social Influence (SI)

Social influence (SI) is defined as a formative construct of behavioral intention as the degree to which a person perceives that other persons that are important to them believe that they should use the particular technology. Several studies have concluded that SI significantly influences technology adoption [44,47,48]. Therefore, we hypothesized that:

**H3:**  *Social influence (SI) has a statistically significant influence on the behavioral intention to adopt mHealth.*

#### 1.2.4. Self-Concept (SC)

Researchers have recognized an integrated effect of psychological phenomena on individuals’ willingness to adopt any technology. However, individuals’ personalities and internal self-perceptions regarding the significance of any product have an added appeal in conceptualizing another consumer preference component, which is called self-concept [28,49]. Therefore, it is hypothesized that:

**H4:**  *Self-concept (SC) has a statistically significant influence on the behavioral intention to adopt mHealth.*

#### 1.2.5. Facilitating Conditions (FCs)

The concept of facilitating conditions (FCs) is defined as the degree to which an individual believes that the infrastructure, such as organizational and technical, exists for the utilization of mobile health [20]. In the context of mHealth technologies, the factor FCs is considered to be a significant factor. The successful and effective use of mHealth service systems is heavily dependent on uninterrupted contact between the service provider and host, who are in two different locations. Several research studies have predicted FCs to be a significant predictor of technology adoption [42,50,51,52,53]. Thus, we proposed the following hypothesis:

**H5:**  *Facilitating conditions (FCs) is a significant predictor for adopting mHealth.*

#### 1.2.6. Behavioral Intention (BI)

The behavioral intention of an individual refers to their individualistic/subjective probability of carrying a specific behavior [26]. Based on a theoretical perspective, behavioral intention has a significant impact on the use of technology [33,39,54]. Therefore, it is hypothesized that:

**H6:**  *Behavioral intention is a statistically significant predictor of the adoption of mHealth.*

### 1.3. Objectives of the Study

Pakistan is the fifth most populated country in the world, with 64% of its population residing in rural areas. The rural population has inadequate access to healthcare services due to a lack of medical facilities and health infrastructure in the rural areas of the country [55]. In developing countries such as Pakistan, the high cost of transportation for mobility to hospitals or medical emergencies due to the long distances and high cost of fuel is one of the major issues [56]. Thus, the adoption of mHealth for the provision of healthcare resources and services is an achievable, cost-effective, convenient, and more efficient method in low-resource settings. 

Therefore, the present study was conducted with objectives to measure and validate the unified theory of the acceptance and use of technology (UTAUT) as well as to identify the predictors to mHealth technology adoption among healthcare professionals (doctors and nurses) in limited-resource settings. 

## 2. Materials and Methods

### 2.1. Participants and Procedure

A cross-sectional survey was carried out at the six public and private hospitals in the two districts (Lodhran and Multan) of Punjab, Pakistan. The participants of the study comprised registered doctors and nurses working in the participating hospitals. Of these six hospitals, medical colleges are attached to three hospitals (two public and one private) for the provision of undergraduate and graduate medical education and trainings. The other three hospitals are classified as secondary healthcare centers. The population’s characteristics may not vary based on the centers’ characteristics (tertiary or secondary healthcare) due to the requirements of a basic degree (BSN for nursing and MBBS/FCPS for doctors/consultants) recognized by the Pakistan Medical and Dental Council (PMDC) and Pakistan Nursing and Midwifery Council (PNMC) for appointments in medical centers/hospitals. 

### 2.2. Research Tool

A two-part questionnaire was developed after reviewing the relevant literature for assessing the study’s settings and the status of the participants in the healthcare facilities in terms of how health is currently delivered, the status of infrastructure and facilitating conditions, and the need for mHealth in the facilities. The first part of the questionnaire comprised demographics-related questions, such as the respondents’ gender and age, the professionals’ experience and profession (doctor or nurse), and the working unit (emergency, primacy care, medical, or surgical units). The second part comprised seven sub-scales and 34 statements. The first sub-scale, performance expectancy (PC), contained six statements, the sub-scale on effort expectancy (EE) included five statements, and the sub-scales on the facilitating conditions (FCs) and social influence (SI) contained four statements each. However, the sub-scales on self-concept (SC) and behavioral intention (BI) both comprised five statements each. The last sub-scale on mHealth adoption was measured using five statements. 

The questionnaire was pre-tested by three experts from the field of information management, public health, and health communication. The recommended changes, such as shuffling and rephrasing statements, were incorporated in the questionnaire. 

### 2.3. Data Collection and Analysis Procedure

Purposive sampling was used to collect the data. A total of 500 questionnaires were sent out to participants through personal visits to their clinics, sending them emails, and posting them printed copies of the questionnaire. The participants were informed that they could leave the questionnaire at any time and that their participation was completely voluntary. Of the 500 questionnaires, 314 filled questionnaires were returned with a response rate of 62.8% after three follow-ups with a gap of two weeks each. All these 314 copies of the questionnaire were valid for data analysis. The Statistical Package for Social Sciences (SPSS software v26) software was used for the data analysis. The dataset’s missing values were replaced using expectation-maximization (EM) methods. Demographic data are given as percentages and frequencies. The analysis of moment structures (AMOS) method was used for confirmatory factor analysis, structural equation modelling, and multi-group analysis. The confirmatory factor analysis (CFA) method was used to examine the association between the latent variables and model estimates. The structural equation model (SEM) was then used to estimate the direct and indirect effects of various UTAUT model paths and to determine the validation of the hypotheses. The significance value was set at < 0.05. The study was started after approval was obtained from the Departmental Research Committee, Department of Information Management, The Islamia University of Bahawalpur, Pakistan.

## 3. Results

### 3.1. Demographic Information

Of the 314 respondents, 164 (52.2%) were female and 150 (47.8%) were male. The majority (182 (58%)) of the respondents were doctors, and 132 (42%) were nurses. The majority (268 (85.4%)) were less than 35 years of age, while only 13 (4.1%) were over 50 years old. The majority (264 (84%)) of the respondents had less than 10 years of professional experience. A total of 110 (35%) respondents were from primary healthcare, 71 (22.6%) were from medical or surgical units, 55 (17.5%) were from intensive care, 36 (11.5%) worked in an emergency unit, and 41 (13.3%) worked in an operating unit.

### 3.2. Confirmatory Factor Analysis

Cronbach’s alpha was used to assess the questionnaire’s reliability. The six statements for PE received a Cronbach’s alpha score of 0.890, the five-item loading for EE received a score of 0.893, the four items for FCs obtained a score of 0.839, the four items for SI received a score of 0.862, SC received a score of 0.885, the statements on the construct BI received a Cronbach’s alpha score of 0.885, and the five statements for mHealth adoption received a score of 0.673. Cronbach’s alpha value for the questionnaire’s 34 statements over seven constructs was 0.966, indicating strong reliability.

**Figure 2:**
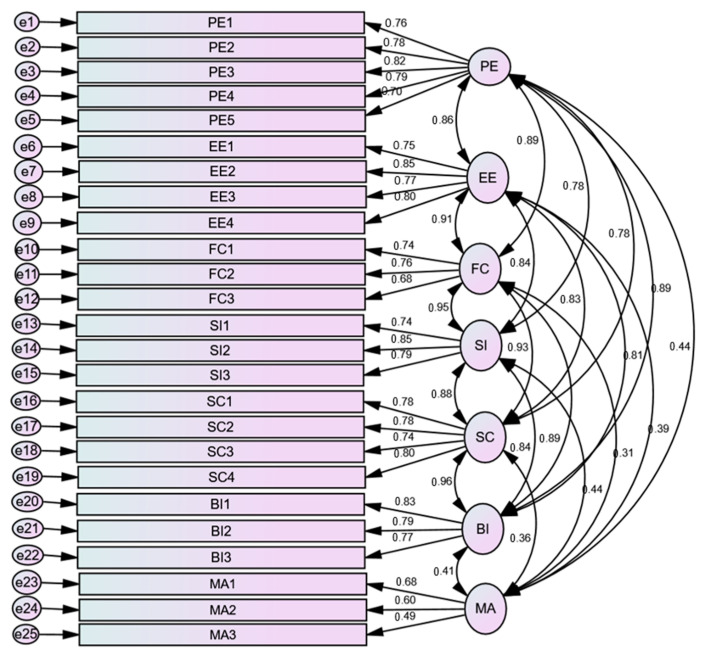
Confirmatory factor analysis (CFA) estimating the constructs. See Appendix A for detailed descriptions of performance expectancy (PE), effort expectancy (EE), facilitating conditions (FCs), social influence (SI), self-concept (SC), behavioral intention (BI), and mHealth adoption (MA).

A seven-factor measurement model for PE, EE, FCs, SI, SC, BI, and MA was estimated by applying confirmatory factor analysis (CFA) (Figure 2). The model fit indices indicated that the model values were acceptable: χ^2^ = 3.206; df = 254; *p* = 0.000; RMSEA = 0.084; CFI = 0.893; IFI = 0.894; and TLI = 0.874. The chi-square value of 3.206, which was slightly greater than the accepted value (<2 or 3) [57], also had *p* < 0.05, suggesting a significant difference between the proposed and the observed models.

#### 3.2.1. Regression Weights 

Figure 2 displays the standardized estimation of the regression weights of the loading of the components on the constructs. The latent variables’ (PE, EE, FCs, SI, SC, BI, and MA) path coefficient values were found to be moderate-to-high, ranging from β = 0.49 to β = 0.85. The latent variable PE was measured using five observable variables, and the loading values ranged between β = 0.70 and β = 0.82, showing strong loadings on the construct. The latent variable EE was measured using four items, and the values ranged between β = 0.75 and β = 0.85, demonstrating a strong correlation between the loadings and the construct. The latent variables FCs, SI, and BI were measured using three observable items on each latent variable. All these items received values that ranged between β = 0.68 and β = 0.85, suggesting a strong correlation between the items and the construct. The score of the four items on SC ranged between β = 0.74 and β = 0.80, indicating a strong association of the loadings. The loading values of the MA items varied from 0.49 to 0.68, indicating a moderate-to-strong association.

#### 3.2.2. Estimation of Correlation

The standardized correlation estimation between the seven latent variables is shown in Figure 2. The correlation coefficient (β =0.86) showed that PE and EE had a strong positive relationship. PE was strongly correlated with FCs (β = 0.89), SI (β = 0.078), SC (β = 0.81), and BI (β = 0.89). The correlation between PE and MA was moderately strong (β = 0.44). On the other hand, we found a strong correlation between EE and FCs (β = 0.91), SI (β = 0.84), SC (β = 0.83), and BI (β = 0.81). However, EE had a moderate correlation with MA (β = 0.39). The factor FCs was strongly correlated with SI (β = 0.95), SC (β = 0.93), and BI (β = 0.89). However, the strength of the correlation between FCs and MA was moderate (β = 0.31). SI was strongly correlated with SC (β = 0.88), BI (β = 0.84), and MA (β = 0.45). There was a moderate-strength positive correlation between BI and MA (β = 0.412). 

### 3.3. Structural Equation Model

**Figure 3:**
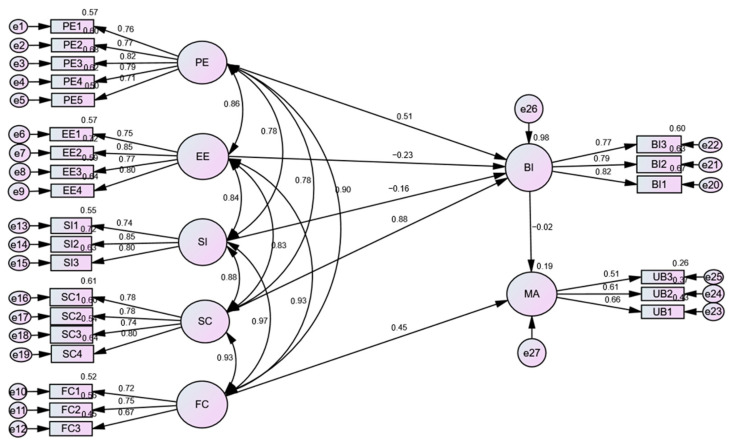
Structural Equation Model.

The structural equation model (SEM) was applied to calculate the effects (direct and indirect) of the constructs of the UTAUT model on the adoption of mHealth (Figure 3). The correlation value indicated that PE was strongly associated with EE (β = 0.86), SI (β = 0.78), SC (β = 0.78), and FCs (β = 0.90). The path coefficient estimation indicated that PE influenced BI (β = 0.51). SC influenced BI (β = 0.88), and FCs influenced MA (β = 0.45). However, EE (β = −0.23) and SI (β = 1.16) negatively influenced BI.

#### 3.3.1. Multiple Squared Correlations

The values of the squared multiple correlations showed that the four modelling factors (PE, EE, SI, and SC), collectively accounted for 98% of the variance in the BI. However, the combined effect of PE, EE, SI, SC, and FCs via BI accounted for 19% of the variance in MA (Figure 3).

#### 3.3.2. Standardized Direct, Indirect, and Total Effects 

In the structural equation model (as shown in Figure 3), BI mediated the effects of PE, EE, SI, and SC on MA. However, there was also a direct path from FCs to MA, as given in the original model (Figure 1). The estimation indicated that SC (β = −0.015) and PE (β = −0.009) had negative indirect effects on MA. Similarly, SI (β = −0.156) and EE (β = −0.230) had a negative direct effect on MA. On the other hand, SC (β = 0.882), and PE (β = 0.514) has positive total effects on the BI (Figure 3).

#### 3.3.3. Model Fit Indices 

Metrics like the incremental fit (IFI, CFI, TLI) and absolute fit (χ^2^, RMSEA) were used to verify the model’s goodness-of-fit. The suggested values were CMIN/df ≤ 3, CFI ≥ 0.90, IFI ≥ 0.90, TLI ≥ 0.90, and RMSEA ≤ 0.08 [57]. The findings indicated that the goodness-of-fit values were within the acceptable range: χ^2^ (df = 259) = 3.207; *p* = 0.000; CFI = 0.891; IFI = 0.892; TLI = 0.874; and RMSEA = 0.084.

### 3.4. Moderating Effect of Gender, Age, and Experience

Using a multi-group analysis, this study examined the moderating effects of age, gender, and experience on the relationship between PE, EE, SI, SC, FCs, BI, and MA. 

#### 3.4.1. Moderating Effect of Age

Our findings showed that the relationship between the UTAUT components was significantly moderated by age (CMIN (DF = 12): 22.505, *p* = 0.032). Age had a significant impact on the relationship between FCs and MA (χ^2^ (df = 2): 8.405, *p* = 0.015) and on the relationship between PE and BI. It also significantly moderated the relationship between BI and MA (χ^2^ (df = 2): 13.401, *p* = 0.001). However, there was moderating influence of age on the relationship between PE and BI (χ^2^ (df = 2): 1.613, *p* =0.446), EE and BI (χ^2^ (df = 2): 3.002, *p* = 0.223), SI and BI (χ^2^ (df = 2): 0.525, *p* = 0.769), and SC and BI (χ^2^ (df = 2): 0.367, *p* = 0.832).

#### 3.4.2. Moderating Effect of Gender

Gender had a statistically significant moderating effect (χ^2^ (DF = 6) 24.380, *p* = 0.000) on the relationship between SI and BI (χ^2^ (df = 1): 16.044, *p* = 0.000) and SC and BI (χ^2^ (df = 1): 20.183, *p* = 0.000). However, we found no significant influence of gender on the relationship between PE and BI (χ^2^ (df = 1): 2.964, *p* = 0.085), EE and BI (χ^2^ (df = 1): 0.958, *p* = 0.328), FCs and MA (χ^2^ (df = 1): 1.578, *p* = 0.209), and BI and MA (χ^2^ (df = 1): 0.1.516, *p* = 0.218).

#### 3.4.3. Moderating Effect of Experience

Experience had a statistically non-significant positive moderating effect (χ^2^ (df = 18) 23.036, *p* = 0.189) on the relationship between PE and BI (χ^2^ (df = 3): 1.365, *p* = 0.714), EE and BI (χ^2^ (df = 3): 1.915, *p* = 0.590), SI and BI (χ^2^ (df = 3): 6.280, *p* = 0.099), FCs and MA (χ^2^ (df = 3): 2.951, *p* = 0.399), and BI and MA (χ^2^ (df = 3): 2.611, *p* = 0.456).

### 3.5. Estimation of Regression Weights and Validation of the Hypotheses 

Table 3 displays the standardized regression estimates, critical ratio (CR), significance of components, standard error (SE) for parameter estimation, and confirmation of hypotheses. Regression analysis can be used as a measurement to predict the variance in one variable depending on another. The level of significance was fixed at *p* = 0.05, and the notation “***” represents the probability that the variable’s value would go below the alpha value of 0.005. The findings indicated that PE significantly influenced BI (β = 0.504, CR = 5.064, *p* < 0.05). Similarly, SC significantly influenced BI (β = 0.860, CR = 5.968, *p* < 0.05). On the other hand, there was significant influence of EE (β = −0.198, CR = −1.900, *p* = 0.057) and SI (β = −0.134, CR = −1.109, *p* = 0.267) on BI, and FCs also had no significant influence on MA (β = 0.219, CR = 1.916, *p* = 0.55). Furthermore, no significant influence of BI was found on MA (β = −0.008, CR = −0.054, *p* = 0.957). 

## 4. Discussion

Our study measured the unified theory of acceptance and use of technology (UTAUT) among healthcare professionals in low-resource healthcare settings. Several studies have already applied the UTAUT model to predict mHealth adoption [58,59,60,61,62,63]. The CFA validation findings of the seven-factor measurement model (PE, EE, FCs, SI, SC, BI, and MA) based on the 25 valid items (9 items with a low loading on the constructs in the model were removed) showed a strong correlation between PE and EE, PE and FCs, PE and SI, PE and SC, and PE and BI. These findings are comparable with previous studies [64]. The correlation between PE and MA, however, was found to be only moderately strong. 

On the other hand, we found a strong correlation between EE and FCs, SI, SC, and BI. However, it had a moderate-level correlation with MA. However, the model indicated that the factor FCs was strongly correlated with SI, SC, and BI. However, the strength of the correlation between FCs and MA was moderate. Our findings validate the findings of a previous study that reported that SI is strongly correlated with SC, BI, and MA [42]. SC was also positively correlated with BI and MA. There was a moderate-strength positive correlation between BI and MA. However, BI was a strong predictor for MA. The literature also shows that a higher BI level predicts the actual use behavior of mHealth adoption [40,61,65]. Overall, the goodness-of-fit values showed that the CFA model was acceptable (χ^2^ = 3.206; df = 254; *p* = 0.000; RMSEA = 0.084; CFI = 0.893; IFI = 0.894; and TLI = 0.874). 

Structural equation modeling (SEM) was applied to validate the hypotheses of this study. The correlational scores indicated that PE was strongly associated with EE, SI, SC, and FCs. The path coefficient estimation showed that PE was positively corelated with BI. Our findings validate the findings of previous studies that reported a strong correlation between PE and BI [59,60,62,63]. However, in contrast to our findings, [53] claimed that PE does not significantly influence BI.

Our findings support the findings of another study that reported that SC influences BI and the factor FCs influences MA [66]. However, EE and SI negatively influence BI. These results are in contrast with the findings of previous studies showing a positive influence of SI on BI in the context of mHealth [42].

The goodness-of-fit values of the SEM model were found to be acceptable (χ^2^ (df = 259) = 3.207; *p* = 0.000; CFI = 0.891; IFI = 0.892; TLI = 0.874; and RMSEA = 0.084). Overall, the model fit indices validated the UTAUT model in our study’s population. 

### 4.1. Moderating Effect of Gender, Age, and Experience

The findings of our study showed that the relationships between the UTAUT components (PE, EE, FCs, SI, SC, BI, and MA) were significantly moderated by age. These findings are comparable with a study [67] that showed that age has a significant impact on the relationship between FCs and MA and that the relationship between PE and BI is influenced by age. It also moderates the relationship between BI and MA. However, our study did not find a moderating influence of age on the relationship between PE and BI, EE and BI, SI and BI, and SC and BI.

Our findings indicated that gender had a statistically significant moderating effect on the relationship between SI and BI as well as on the relationship between SC and BI. Likewise, several previous studies have reported gender to be a key moderator of mHealth adoption [68,69]. However, there was significant moderating influence of gender on the relationship between PE and BI, EE and BI, FCs and MA, and BI and MA. Previously, similar findings have shown that gender is not a key moderator of the adoption of mHealth services [62]. 

Our results indicated that experience had a statistically non-significant positive moderating effect on the relationship between PE and BI, EE and BI, SI and BI, FCs and MA, and BI and MA. 

The findings of our study statistically validated only two of the study’s six hypotheses, indicating that PE and SC had a statistically significant influence on the BI of healthcare professionals towards mHealth adoption. Previous studies have also indicated a positive influence of PE on BI [40,70,71]. The UTAUT model comprised six components (PE, EE, SI, FCs, BI, and MA). However, we added an extra component, SC, with the help of the literature in order to enhance the model’s strength and to verify whether SC has an influence on behavioral intentions. The findings of our study showed that SC has a significant influence on BI. However, a previous study indicated a non-significant influence of SC on BI [64]. Furthermore, our study indicated that the factor FCs has a positive influence on MA, but the influence is not statistically significant. On the other hand, EE and SI have a non-significant negative influence on BI. Similarly, BI has a negative non-significant influence on MA among healthcare professionals.

### 4.2. Limitations of the Study

We used a survey method for data collection, which is always subject to respondents’ own understanding and self-reporting in terms of the question statements. Therefore, there is always a chance that the respondents’ answers to the question statement may differ from the actual conditions. In order to minimize the limitations of the questionnaire, the questionnaire was pre-tested by three experts from the field of information management, public health, and health communication. Cronbach’s alpha was used to assess the questionnaire’s reliability. The 34 items under seven constructs received a Cronbach’s alpha value of 0.966, showing the high reliability of the questionnaire. Furthermore, we acknowledge the Dunning–Kruger effect (DKE) [72,73] as a limitation of the questionnaire given that it relies on subjective self-reporting. DKE is a cognitive bias that causes individuals to overestimate their abilities. Sometimes, we think (overestimation) that we know or understand something well but we actually do not (‘being ignorant of one’s own ignorance’). The authors also acknowledge the potential selection bias for study’s population and healthcare centers for data collection as a limitation of the study. Therefore, care should be exercised while generalizing the findings of this study to the population of secondary- or tertiary-care hospitals. However, we adopted purposive sampling in order to avoid selection bias, but it is inevitable and fundamental in this type of survey study. 

The application of international regulations by the various governments is one of the requirements for mHealth perceivability. Regulatory and legal issues/frameworks are important when considering mHealth apps/services adoption, especially apps/services that behave as a medical device (SaMD or software as a medical device) [74]. However, the regulatory and legal aspects of mHealth adoption are not discussed in this study, which may be a limitation of this study. 

### 4.3. Implications of the Study

This study has several practical implications. Ensuring universal health coverage is a part of the United Nations’ sustainable development goals. The SDG 3 “Good health and well-being” attempts to promote healthy lives at all ages. Pakistan is a country with low resources; it has an estimated population of 240 million people, and almost 64% of population lives in rural areas with poor health indicators due to limited access to health resources and services [55,56]. In this context, the adoption of mHealth can significantly help in improving people’s access to quality healthcare resources and services and in reducing costs. It is notable here that the information communication and technology infrastructure is quite good in Pakistan, where there are an estimated 191 million mobile cellular subscribers.

The findings of this study have implications for policy makers, as it identified that performance expectancy and self-concept are the main predictors for mHealth adoption among healthcare professionals. Therefore, there is a need for policy makers to demonstrate the capabilities of mHealth in transforming healthcare delivery across rural and urban areas. There is also a need to conduct hands-on sessions and awareness programs in order to demonstrate the performance of mHealth technologies and to develop self-concept about mHealth technologies and its capabilities among healthcare professionals. The way forward is to launch an mHealth pilot in each district. The success of these pilots will involve the whole community of healthcare professionals for mHealth adoption. The involvement of peers and influential people, such as people who are policy makers, early mHealth adaptors, or successful health care professionals, can play a significant role in the adoption of mHealth among healthcare professionals at the level of critical mass. 

The speed of mHealth adoption is quite slow in Pakistan. The socio-economic and low literacy level are among the main reasons that prevent the adoption of mHealth in Pakistan [7]. Moreover, in developing countries such as Bangladesh, Pakistan, and India, the prevalence of traditional culture, the digital divide, the lack of technical skills, and poor health-related information-seeking behaviors all contribute to technology anxiety and resistance to adopting new technologies like mHealth [75]. The acceptance of mHealth capabilities would increase exponentially if people believe that mHealth is practical and convenient and could contribute to accessing healthcare. Therefore, there is a need to improve the mobile health literacy of the general population so that they can use mobile health applications for locating relevant doctors, making doctor’s appointments, seeking advice from doctors, receiving telemedicine, conducting patient-to-doctor conversations through video, and receiving prescriptions. Governments’ health departments have a key role in improving mobile health literacy, and they could achieve this with the involvement of health experts, health educators, and technology experts. The general population can be given awareness and demonstration sessions at hospitals. Moreover, an awareness video explaining the steps involved in the use of mHealth services can also be disseminated among the general population. This study recommends the need for improving the mobile health literacy of the general population. Furthermore, it is important to design user-friendly mobile health applications that will reduce the likelihood of non-adoption. Jacob et al. [76] recently proposed a shift toward theoretical frameworks that address implementation challenges, taking into account the complexity of the sociotechnical structure of healthcare organizations as well as the interplay between technical, social, and organizational aspects.

## 5. Conclusions

This study validates the unified theory of the acceptance and use of technology (UTAUT) model with an additional construct of self-concept among healthcare professionals (doctors and nurses) in low-resource environments. It concludes that performance expectancy and self-concept are the main predictors that influence the behavioral intention of healthcare professionals towards mHealth adoption, while age and gender are moderating factors in terms of mHealth adoption. This study suggests that the adoption of mHealth can significantly help in improving people’s access to quality healthcare resources and services, reducing costs and health disparities as well as promoting health in low-resource settings.

## Figures and Tables

**Table 1:** Type of Services.

Types of Service	Description
- Awareness and education	- Awareness raising - Health telephony - Emergency number—free telephone numbers - Health promotion and community mobilization - Informational programs
- Remote data gathering	- Patient records - Health surveys and surveillance
- Remote data monitoring	- Reminders for appointments - Monitoring of patients - Compliance with treatment
- Healthcare worker training and communication	- Mobile telemedicine
- Tracking of disease and epidemic outbreaks	- Public health emergencies - Health checks and monitoring
- Support for diagnostics and treatment	- Patient records for the decision-supporting telemedicine system

**Table 2:** Popular mHealth applications in Pakistan.

Types of App	Description
Marham	The healthcare application Marham allows communication between patients and physicians. The app’s users can use it to locate doctors, make appointments, and obtain medical guidance. It also provides information about hospitals and medical specialties. It has over one million users. https://play.google.com/store/apps/details?id=controllers.marham.marhammed&pcampaignid=web_share (accessed on 16 November 2023)
MyDoctor.pk	The MyDoctor.pk app gives access to a network of doctors and enables them to schedule appointments, communicate with doctors, and examine their medical records. It has over ten thousand users. https://play.google.com/store/apps/details?id=com.doctor.findmydoctor&pcampaignid=web_share (accessed on 16 November 2023)
Oladoc	The Oladoc platform allows users to search for and schedule appointments with doctors, dentists, and other healthcare providers. It has over one million users. https://play.google.com/store/apps/details?id=com.mediconnect.oladoc&pcampaignid=web_share (accessed on 16 November 2023)
Pak Blood	Pak Blood helps people in finding nearby blood donors during emergencies. Through the app, users may connect with potential donors and request blood donations. It has over ten thousand users. https://play.google.com/store/apps/details?id=com.bloodbank.bloodbankpakistan&pcampaignid=web_share (accessed on 16 November 2023)
Pregnancy Week by Week	Pregnancy Week by Week provides counseling, health information, and a week-by-week pregnancy tracker to help pregnant women have a healthy pregnancy. It has over ten million users https://play.google.com/store/apps/details?id=com.hp.pregnancy.lite&pcampaignid=web_share (accessed on 16 November 2023)
Pharmapedia Pakistan	Pharmapedia is a medical app that provides information about medicines that are available in Pakistan, including uses, dosages, and adverse effects. It has over one million users. https://play.google.com/store/apps/details?id=com.binops.pharma.pk&pcampaignid=web_share (accessed on 16 November 2023)
Dawaai.pk	The Dawaai.pk app allows users to buy prescription medicines, healthcare products, and supplements along with additional information regarding medications and their substitutes. It has over one million users. https://play.google.com/store/apps/details?id=com.dawaai.app&pcampaignid=web_share (accessed on 16 November 2023)

**Table 3:** Validation of Hypotheses.

Hypotheses				Estimate	S.E.	C.R.	*p*	Results
H_1_	PE	→	BI	0.504	0.099	5.064	***	Accepted
H_2_	EE	→	BI	−0.198	0.104	−1.900	0.057	Rejected
H_3_	SI	→	BI	−0.134	0.121	−1.109	0.267	Rejected
H_4_	SC	→	BI	0.860	0.144	5.968	***	Accepted
H_5_	FCs	→	MA	0.219	0.114	1.916	0.055	Rejected
H_6_	BI	→	MA	−0.008	0.153	−0.054	0.957	Rejected

The symbol ‘***’ denotes the likelihood that the variable’s value will not exceed the critical threshold of 0.005. All hypotheses’ abbreviations are expanded in Appendix A.

## Data Availability

The core data supporting the findings of this study are available within the article and Appendix A; further details can be obtained from the authors upon reasonable request.

## Appendix A. Items under Seven Constructs of the UTAUT

**Construct**	**Items**	**Measure**	**Sources**
**Performance Expectancy (PE)**	PE1	I find mHealth useful in my daily life.	[26,29,77]
	PE2	I think mHealth can help in providing timely medical advice to patients	
	PE3	I think mHealth can help in accomplishing daily tasks more quickly.	
	PE4	I think adopting mHealth can increase my clinical performance.	
	PE5	I think mHealth can reduce a considerable amount of hospital expenses on the provision of healthcare	
	PE6	I think mHealth application allows healthcare professionals to be easily accessible and thereby enhances patient experience.	
**Effort Expectancy (EE)**	EE1	I think learning how to monitor health issues through mHealth is easy	[33]
	EE2	I think my interaction with patients through mHealth app is clear and understandable	
	EE3	I think mHealth apps are easy to use	
	EE4	I think I do not need any effort to use mHealth for patient care	
	EE5	I think it is easy to learn how to use mHealth to receive, track, and evaluate medical data	
**Social Influence (SI)**	SI1	People who influence my behavior think that I should use mHealth for clinical practice	[42]
	SI2	People whose opinions that I value prefer that I use mHealth for clinical practice	
	SI3	Professionals in my community who use mHealth are more informed and active than those who do not	
	SI4	My mentors and senior professionals encourage me to use mHealth for providing patient care.	
**Self-Concept (SC)**	SC1	I think the application of mHealth is necessary for clinical practice	[64]
	SC2	I think there is strong evidence that supports the use of mHealth in order to improve the quality of patient care	
	SC3	I feel adherence to mHealth characteristics.	
	SC4	I think my personal behavior is compatible with the mHealth image.	
	SC5	I think mHealth can help in monitoring patients, issuing diagnoses, and treating diseases.	
**Facilitating Conditions (FCs)**	FC1	I think I have access to the secured and trusted resources necessary to practice mHealth.	[27,77,78]
	FC2	I think I have gathered the knowledge necessary to use mHealth.	
	FC3	I think I can get help from my colleagues when I have difficulties using mHealth.	
	FC4	I think our hospital encourages and facilitates the use of mHealth	
**Behavioral Intention (BI)**	BI1	I intend to use mHealth in the future	[79]
	BI2	I am interested in r improving the skills necessary to incorporate mHealth into clinical practice	
	BI3	If I need to check patients from rural areas for health-related issue, I intend to use mHealth.	
	BI4	If mHealth brings convenience to my patients, I am willing to continue using it	
	BI5	I intend to inform my colleagues and other healthcare professionals to use mHealth	
**mHealth Adoption for Healthcare**	AD1	I think adopting mHealth service will be a pleasant experience	[63,65,67,80]
	AD2	I think mHealth can provide an opportunity to respond patients more quickly	
	AD3	I spend a lot of time on using mHealth applications	
	AD4	I think adapting mHealth can enable faster access to patient data.	
	AD5	I frequently use mHealth services.	
